# Plant growth regulator extracts from seaweeds promote plant growth and confer drought tolerance in canola (*Brassica napus*)

**DOI:** 10.1080/15592324.2023.2267222

**Published:** 2023-10-30

**Authors:** Justin B. Nichol, Alynne Kris B. Ribano, Neil M.N. Hickerson, Nusrat Ali, Frank Jamois, Marcus A. Samuel

**Affiliations:** a Department of Biological Sciences, University of Calgary, Calgary, Alberta, Canada; b Phys-Chem and Bio-Analytics Department, Agro Innovation International, Centre Mondial de l’Innovation Roullier - TIMAC AGRO, Saint-Malo, France

**Keywords:** Seaweed extract, biostimulant, growth and development, drought, transcriptomics

## Abstract

*Brassica napus*, commonly known as canola, is an important oilseed crop in Canada contributing over $29.9 billion CAD to the Canadian economy annually. A major challenge facing Canadian canola is drought, which has become increasingly prevalent in recent years due to the changing climate. Research investigating novel agronomic techniques in mitigating drought is key to securing yields and sustainability in canola and other crops. One such technique is the use of bio-stimulant sprays to help offset biotic and abiotic stresses in plants through promoting stand establishment. Previous studies have shown that the application of seaweed extracts as bio-stimulant sprays to Brassicaceae has been successful in improving plant growth and development along with stress tolerance. However, this method has yet to be tested on canola. The organic nutrients that are waste products from processed seaweed help stimulate plant growth, yielding higher quality plants as a result. In association with Le Groupe Roullier, this study demonstrates that the Roullier extracts (RE) help increase plant growth characteristics and drought tolerance in canola when sprayed 3 times over a 3-week period. A high yielding but drought sensitive mutant of canola, d14 (developed through gene editing) was used for drought assays after 8 weeks of growth and where water was withheld for 6 days. Application of the REs prior to drought resulted in plants having enhanced survival rate and improved biomass retention indicating high drought tolerance. Subsequent RNA sequencing and gene ontological term analysis performed using RE treated plants in triplicates, revealed substantial levels of differential expression of growth-related genes along with stress-related genes. These REs elicited responses in plants that had previously only been achieved through gene editing and transgenic methodologies. Using bio-stimulant sprays provides a novel platform to promote beneficial agronomic traits, independent of genetic manipulation.

## Introduction

*Brassica napus*, commonly known as canola, is an important oilseed crop in Canada contributing over $29.9 billion CAD to the Canadian economy annually.^1^ A major challenge facing Canadian canola is a reduction in yield and difficulty in crop establishment due to heat and water stress, which has been increasingly prevalent in recent years due to the changing climate.^2,3^ Particularly, drought substantially impacts the morphological and physiological processes in canola.^3^ Therefore, mitigating the effects of drought and other abiotic stressors will be key to generating higher yields and increasing the sustainability of canola.^2^

Use of seaweed extracts as bio-stimulant sprays to help offset biotic and abiotic stresses in plants is a technology that has gained significant attention recently.^4,5^ Bio-stimulant sprays have been shown to increase growth characteristics, including shoot biomass, flowering, yield, growth, and quality of crops. It is speculated that one mode of action is how these sprays can act specifically as elicitors to induce enzymatic activity, thereby promoting nutrient uptake.^4,6–8^ Seaweed-based bio-stimulant sprays offer a novel alternative method to promote beneficial agronomic traits, independent of genetic manipulation, and can lead to reduced synthetic fertilizer use.^4,9^ Although previous research has shown that the application of seaweed extracts as bio-stimulant sprays in Brassicaceae can efficiently increase growth characteristics, this approach has not been tested on canola.^5^

In collaboration with Agro Innovation International – TIMAC AGRO, a blind study was conducted to investigate five Roullier Plant Growth Regulator Extracts (RE1, RE2, RE3, RE4, and RE5) prepared by Agro Innovation International (Saint-Malo, France) for the current investigation. RE1 and RE2 are seaweed-based extracts, while RE5 is a mineral-based extract. RE3 is a combination of RE1 and RE5, whereas RE4 is a combination of RE2 and RE5. The chemical formulation of the Roullier Plant Growth Regulator Extracts prepared and used within this study are confidential. In this study, a treatment regimen conducted under a controlled environment was established to test the efficiency of these extracts in mediating growth characteristics such as promoting traits associated with stand establishment as well as the ability to improve drought tolerance in canola. Our results provide conclusive evidence that these extracts are effective in promoting biomass accumulation and can simultaneously prime the plants to tolerate drought.

## Results and discussion

### RE treatments promote growth and reproductive potential

**Figure 1.**
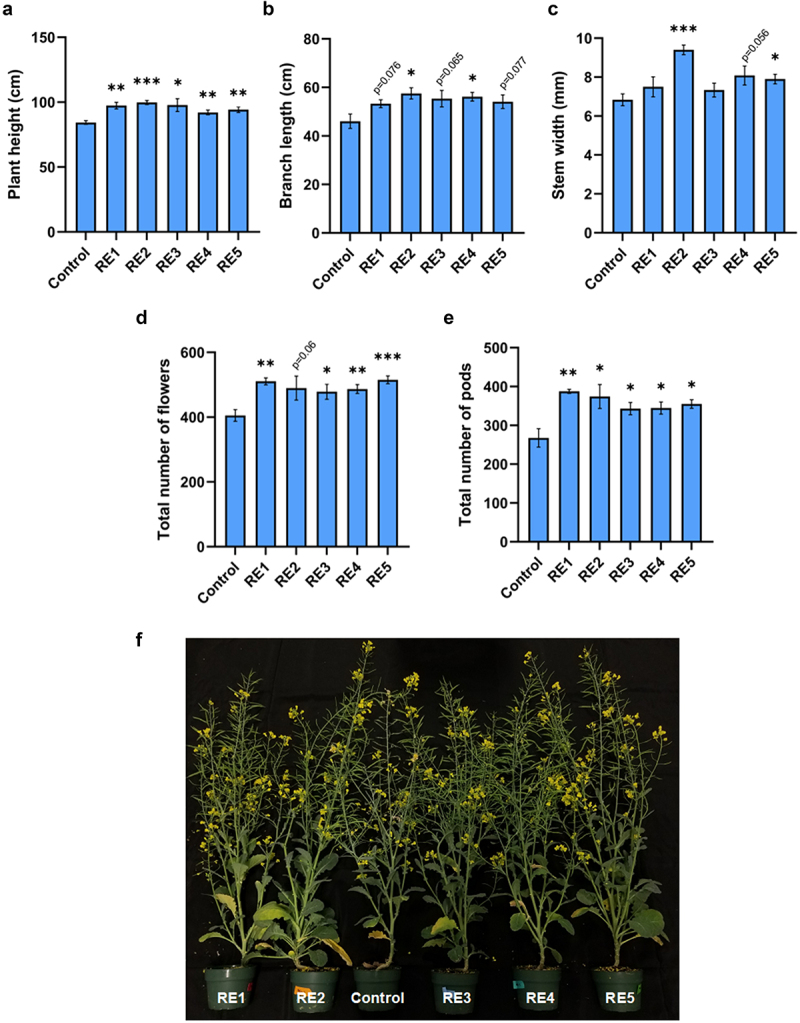
Effect of RE1–5 bio-stimulant sprays on wild-type (Westar, *Brassica napus*) canola growth and reproductive metrics: plant height (*n* = 5) (a), branch length (*n* = 5) (b), stem width (*n* = 5) (c), total number of pods (*n* = 4) (d), and flowers (*n* = 4) (e). Representative image of treatment effect at 11 weeks of growth (f). Values reported are means (bars indicate ± SEM). Asterisks indicate significant differences to control determined by Student’s T-test (* = *p* < .05; ** = *p* < .01; *** = *p* < .001).

To determine whether the RE chemistries had an effect on plant growth characteristics, wild-type canola plants were sprayed three times, once per week from week three (6-leaf stage) until week 5 which coincided with the initiation of bolting. Both vegetative and reproductive growth characteristics were quantified during the flowering stages and following complete maturation of the treated plants. To quantify changes in shoot growth, parameters such as plant stem width, plant height, and branch lengths were measured in the treated samples compared to the control mock (water +0.01% SILWET). SILWET 1–77 was used to help reduce the surface tension of both the RE treatments and control mock sprays for increased plant uptake. In general, RE treatment resulted in a significant increase in plant height (Figure 1a), while only RE2-treated plants exhibited a substantial increase in both branch length and stem width, while RE4 and RE5 treatments led to marginal but significant improvements in branch length and stem width respectively (Figure 1b, c). This suggests that RE 2,4 and 5 have the greatest effect when observing changes in growth patterning, with RE2 displaying the greatest difference among all treatments (Figure 1a–c). The total number of flowers increased with all the RE chemistries, nearly all having a significant increase when compared to the control with only RE2 failing to meet the cutoff (*p* = .06) (Figure 1d). More importantly, when number of productive pods were counted, all RE chemistries were found to significantly increase the total number of pods when compared with the control (Figure 1e). RE1 was observed to have an increase of 44.77%, the largest increase seen out of all RE chemistries tested (Figure 1e). This data suggests that RE chemistries help promote functions important for reproductive growth (Figure 1f). Our findings demonstrate that RE2, 4, and 5 had the most profound effect on vegetative growth characteristics and further promoted reproductive output through an increase in total flowers and harvestable pods. The increased stem thickness observed in with RE2 and RE5 treatments is favorable to confer strength and prevent lodging, thereby protecting the plant yield.

### RE sprays induce specific molecular signatures in plants

**Figure 2.**
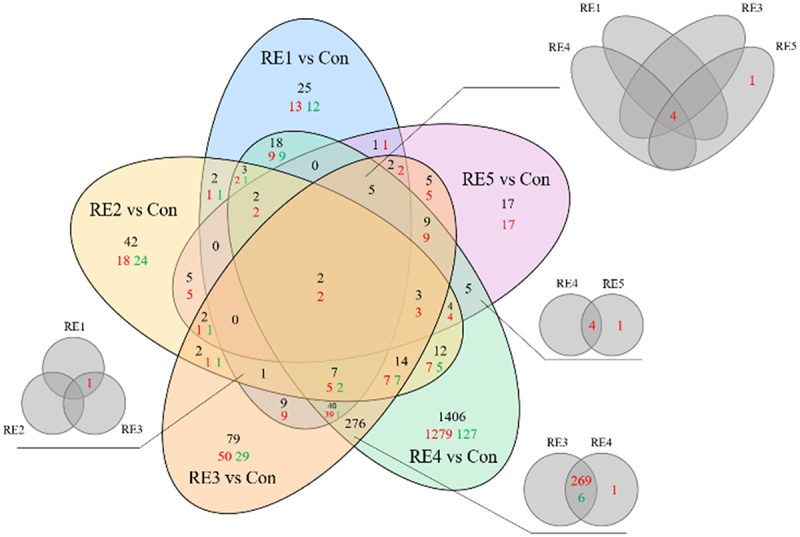
Venn diagram depiction of differentially expressed genes following RE treatments. Whole plant tissue was collected from wild-type canola plants (Westar, *Brassica napus*) treated with RE1–5 after 3 sprays each. Triplicate RNA samples were isolated from each treatment (control and RE1–5) and subjected to RNA sequencing. A cutoff of the absolute value of the Log_2_ fold change ≥ 2 was used to determine differentially expressed genes relative to control plants. The above venn diagram depicts the number of all differentially expressed genes between the RE1–5 treatments (values in black) with an additional breakdown for directionality (up-regulated genes are shown in red, and down-regulated genes are shown in green). Additional venn diagrams demonstrate the overlapping regions for genes with different directionality under different RE treatment conditions.

**Figure 3.**
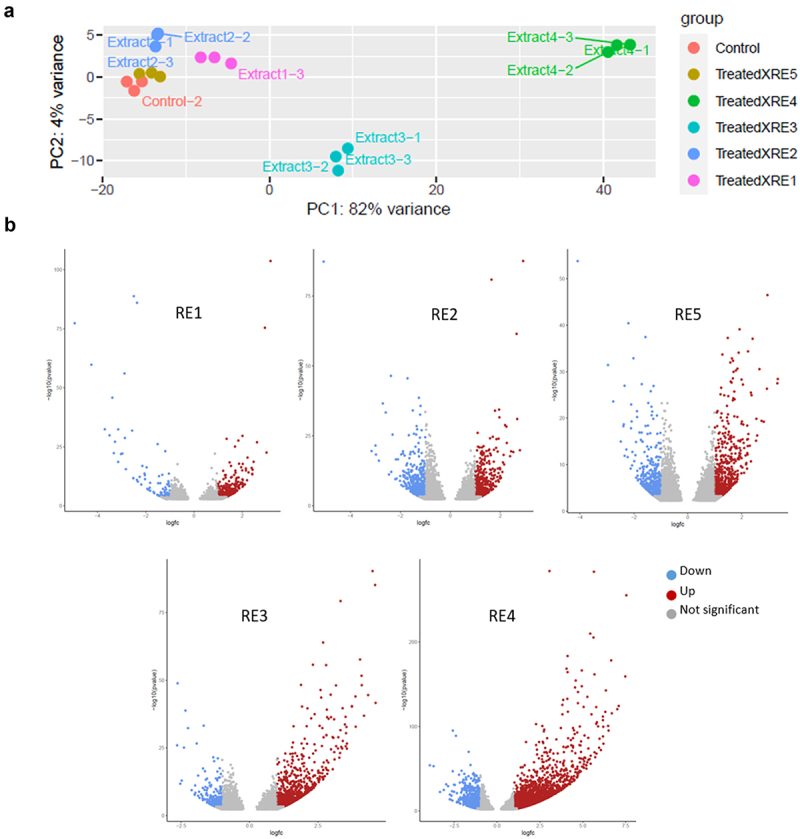
PCA plot depicting the clustering of the triplicate RNA samples isolated from each treatment (control and RE1–5) (a). The above volcano plot represents a graphical illustration of the increasing number of up-regulated genes resulting from the combination of RE1 + RE5 and RE2 + RE5 into RE3 and RE4 respectively.

To further discern the differences in the molecular signatures elicited by these chemistries, we performed RNA sequencing to aid in identifying differentially expressed genes compared to untreated controls. RNA isolated from whole plant tissue was collected in triplicates from wild-type canola plants treated with (RE1–5) after 3 sprays each and compared to mock treated controls through RNA sequencing by the Illumina NovaSeq Platform (Novogene). Differentially expressed genes (DEGs) were chosen using an absolute value of the Log_2_ fold change cutoff of ≥ 2 compared to untreated samples. Interestingly, we observed 1406 differentially expressed genes unique to RE4, with 1279 being up-regulated (Figure 2) suggesting that the RE4 chemistry induces genes in a unique fashion compared to the other extracts tested. Additionally, 276 differentially expressed genes were shared between RE3 and RE4 treatments, with 269 being co-up-regulated (Figure 2) suggesting that treatments RE3 and RE4 trigger a similar suite of genes to promote growth more efficiently since they are combinations of RE1 + RE5 and RE2 + RE5 respectively. The combinatorial effects of the RE sprays led to unique clustering characteristics with RE1, 2, and 5 clustering together, where the combinatorial sprays showed greater variation (Figure 3a). We consistently observed that the use of the RE sprays tended to primarily promote the upregulation of genes, indicating a stimulatory role for genes regulating growth (Figures 2 and 3b).

To obtain a better understanding of the implications of the DEGs, the Plant Gene Set Annotation Database (PlantGSAD) web application tool was used for gene ontological (GO) terms analysis^10^, and KOBAS version 3.0^11^ was used for Kyoto Encyclopedia of Genes and Genomes (KEGG) enriched pathways. In GO, three parts of functional groups including biological process, cellular components, and molecular functions were analyzed. The Fischer exact p-value ≤.05 was considered strongly enriched. Among the different extracts (RE1–5) the most significant GO terms for the biological process were oxidation-reduction process (GO:0055114), photosynthesis (GO:0015979), cellular iron ion homeostasis (GO:0006879), and transmembrane transport (GO:0055085). For the cellular component GO terms, the DEGs for RE1 and RE3 were mostly enriched integral to the membrane (GO:0016021), while RE5 was mostly associated with nucleosomes (GO:0000786). RE2 and RE4 both were mostly enriched in the thylakoid membrane (GO:0042651) and photosystem I reaction center (GO:0009538). The gene ontology molecular function revealed the involvement of DEGs in oxidoreductase activity (GO:0016491), chlorophyll-binding (GO:0016168), transmembrane transporter activity (GO:0022857), and transporter activity (GO:0005215). The KEGG pathway enrichment analysis revealed that the DEGs were associated with specific pathways depending on the extracts (RE1–5). The most significant pathways were glutathione metabolism (bna00480), photosynthesis (bna00195), photosynthesis – antenna proteins (bna00196), 2-oxocarboxylic acid metabolism (bna01210), and glucosinolate biosynthesis (bna00966). These results demonstrate that the GO terms and KEGG pathways that are being primarily enriched following the application of the different extracts (RE1–5) are associated with photosynthetic processes that could lead to increased plant biomass, which we observed within the treated plants as mentioned previously (Figure 1). We also observed an increase in GO terms and KEGG pathways associated with plant growth and development.

**Figure 4.**
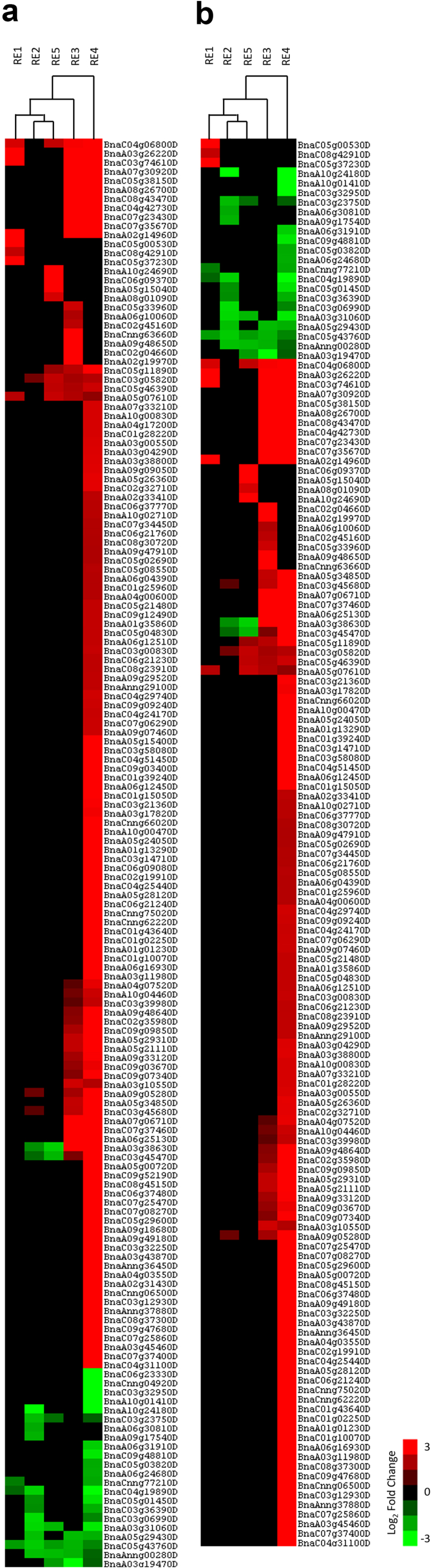
Heat map depiction of differentially expressed genes following GO-term association. Heat maps display expression differences relative to control between RE1–5 based on (a) “non-developmental growth” (GO:0048590; # of genes assessed) and (b) “Abscisic acid-activated Signaling pathway” (GO:0009738/GO:0009787) and “water deprivation” (GO:0048590; # of genes assessed). A unique gene list was used based on all up- and down-regulated genes with the absolute value of Log¬2 fold change ≥ 2.

In order to more specifically classify the genes that were differentially expressed, a unique gene list was created using the previously described cutoffs to identify all up- and down-regulated genes in RE1–5. Using this unique list, we investigated 3 GO terms that were found through the RNA sequencing results. The GO term “Non-developmental Growth” (GO:0048590) was chosen alongside “Abscisic Acid-activated Signaling pathway” (GO:0009738/GO:0009787) and “Water Deprivation” (GO:0048590) to identify a potential molecular basis of growth stimulation as well as to identify any indications of the impact of the REs on stress tolerance. The latter GO terms were combined due to the similarity between ABA signaling and water deprivation pathways. The consequent heat maps for “Non-developmental Growth” indicated a large number of up-regulated genes unique to the RE4 treatment (Figure 4a). Interestingly, RE2 showed primarily down-regulated genes with only a few being up-regulated (Figure 4a). A similar trend in up- and down-regulated genes was observed in the “Abscisic Acid-activated Signaling pathway” and “Water Deprivation” ontological analysis, with RE4 having 113 up-regulated genes and RE2 being mostly down-regulated (Figure 4b). The substantial number of up-regulated genes for RE4 indicates a strong involvement for both of the gene ontologies investigated.

Despite showing considerable morphometric similarity to RE4-treated plants, independent treatments with RE2 or RE5 had very little effect on gene regulation within the chosen GO terms. This strongly suggests that the combinatorial effect of RE2 and RE5 within the RE4 is likely the cause for an effective increase in the regulation of drought associated genes. Furthermore, RE4 treatment uniquely impacted the expression of many ABA/drought-mediated genes and suggested that the application of RE4 could positively impact plant tolerance to drought stress.

### RE treatments confer drought tolerance

**Figure 5.**
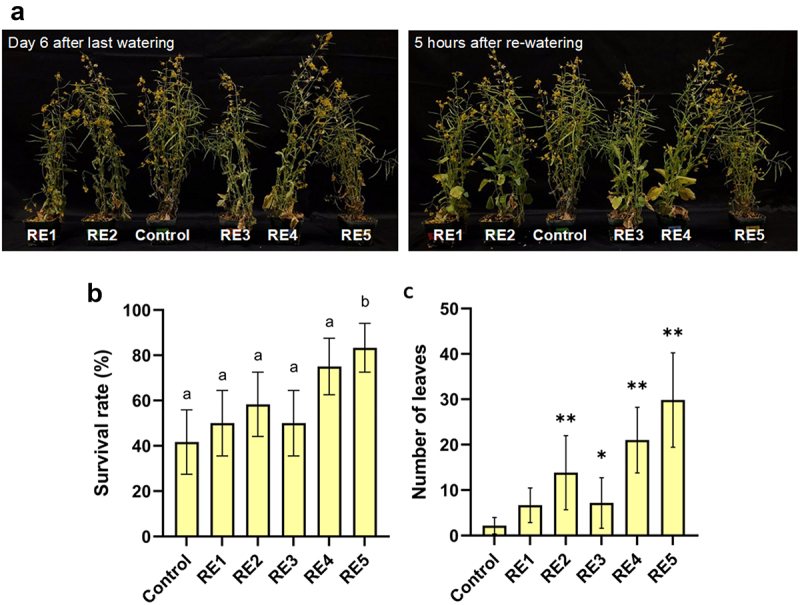
Effect of RE1–5 bio-stimulant sprays on the ability of drought-sensitive *d14* (*Brassica napus*) canola to recover from drought within 5 hours of re-watering (a). (b) the survival rate is reported as a percentage per treatment following drought (*n* = 12, bars indicate ± standard error of proportion). Values with different letters indicate significant differences determined by Z-score for proportions (*p* < .05). (c) number of leaves of survivors (*n* = 6). Values reported are means (bars indicated± SEM). Asterisks indicate significant differences to control determined by Student’s T-test (* = *p* < .05; ** = *p* < .01).

Based on our findings of up-regulation of genes associated with ABA and water deprivation GO terms from our analysis of RNA sequencing data, we investigated whether the RE chemistries could confer drought tolerance. A drought-sensitive canola germplasm developed through gene-editing of the strigolactone receptor (*d14*) was used for the drought assays to allow for greater contrast in drought conditions between treatments.^12–14^^15,16^ After 8 weeks of growth, 72 plants (12 control plants and 12 per 5 RE treatment, which were sprayed 3 times, once per week, from weeks 3 to 5) were subjected to a severe drought by withholding water for 6 days. After 6 days, the plants were re-watered and plant response was captured 5 h after re-watering (Figure 5a). Remarkably, every single RE treated plant was able to recover within 5 h of re-watering while the mock-treated controls could not recover (Figure 5a). This observation strongly suggests that the biostimulant sprays confer protective traits that allow these plants to reestablish quickly after a prolonged period of drought. Other than qualitative observations, indicators used as a baseline for drought tolerance were survivorship percentage and number of viable leaves. RE5 treatment was able to significantly improve plant survivorship following severe drought treatment with 83.3% survival compared to 40% survival of untreated plants (Figure 5b). Application of the RE sprays resulted in a substantial difference in the number of retained viable leaves, with RE2, 3, 4, and 5 showing a significant increase compared to untreated control (Figure 5c). RE4 and RE5 had the strongest effect on viable leaf retention compared to control, with an average of 21 and 29.83 leaves respectively (representing an increase of ~ 9.7–13.8X) compared to control plants with an average of 2 leaves (Figure 5c). This suggests that RE4 and RE5 play a pivotal role in protecting plants under severe drought conditions.

This preliminary study has aided in demonstrating the usefulness of RE chemistries as a bio-stimulant spray for improving canola plant growth, reproduction, and tolerance to abiotic stress. Specifically, the independent extracts RE2, RE5, and their combination RE4 treatments show the greatest impact on vegetative plant growth, reproductive output, survivorship, and maintenance of plant productivity under stress. Given the successful and promising results observed under greenhouse conditions, we are currently employing these sprays under field conditions to assess their efficacy. Through this study, we have been able to conclusively demonstrate that the biostimulants used in this study could significantly improve agronomic traits of critical importance. The added bonus would be that RE treated plants will be primed for drought tolerance, which could serve as crop insurance during seasons with severely water limiting conditions.

In conclusion, from this study, we have been able to unravel the importance of detailed and combinatorial analysis of both individual seaweed extracts as well as their mixed formulations with mineral-based extracts to identify the most effective combination for our crop of interest. In this work, we found that the combination extract RE4 was most effective for its role in promoting growth and enhancing tolerance to drought. RE4 also elicited differential expression of a unique set of stress tolerance genes which was quite different than RE2 and RE5 which make up RE4. Although several seaweed-based extracts are available, it is crucial to develop a detailed experimental design to evaluate the efficacy of these extracts either alone or in combination and follow this up with a molecular approach such as RNA sequencing to understand the cause for the altered effectiveness. Identification of these beneficial effects of RE sprays and the molecular mechanism behind their potential mode of action, provides a viable, non-GM solution to several agronomic traits that are currently only controlled with GM technologies.

## List of abbreviations

DEGs: Differentially expressed genes
GO: Gene ontological
KEGG: Kyoto Encyclopedia of Genes and Genomes
PlantGSAD: Plant Gene Set Annotation Database
RE: Roullier extracts
